# Echinochrome exhibits anti-asthmatic activity through the suppression of airway inflammation, oxidative stress, and histopathological alterations in ovalbumin-induced asthma in BALB/c mice

**DOI:** 10.1007/s00210-023-02678-0

**Published:** 2023-09-26

**Authors:** Islam Ahmed Abdelmawgood, Noha A. Mahana, Abeer Mahmoud Badr, Ayman Saber Mohamed

**Affiliations:** Zoology Department, Faculty of Science, Cairo University, 12613 Giza, Egypt

**Keywords:** Allergic asthma, Ovalbumin, Echinochrome, NF-κB, Anti-inflammatory, Antioxidant

## Abstract

Asthma is a chronic pulmonary disease with marked infiltrating inflammatory cells and reduced respiratory performance. Echinochrome (Ech) is a dark-red pigment isolated from the sea urchin spines, shells, and ova. It has antioxidant, antimicrobial, and anti-inflammatory properties, but whether it can be used in asthma treatment has yet to be investigated. In this research, we aimed to study the inhibitory actions of Ech on allergic asthma symptoms in mice. Mice were divided into 4 groups (*n* = 8 for each): control, ovalbumin-challenged, and Ech-treated (0.1 and 1 mg/kg). At the end of the experiment, nasal scratching, lung oxidative stress, airway inflammation, and remodeling were assessed. In ovalbumin-challenged BALB/C mice, treatment with Ech significantly decreased nasal scratching, lung oxidative stress, inflammatory cell infiltration, mucus hyperproduction and hyperplasia of goblet cells, IgE levels, and inflammatory cytokines. It also inhibited NF-κB phosphorylation. This is the first study to investigate the immunomodulatory effect of Ech against allergic asthma in mice. According to our findings, we imply that Ech may be utilized as a treatment for allergic asthma.

**Graphical Abstract:**

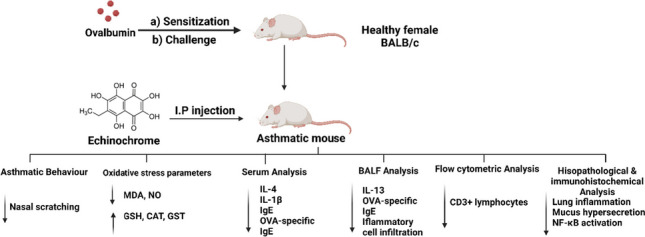

## Introduction

Asthma is a persistent airway condition in which immune dysregulation causes persistent inflammation, resulting in abnormal bronchoconstriction, mucus secretion, and respiratory problems in those suffering (Gillissen and Paparoupa 2015). Asthma is characterized by chronic airway inflammation caused by infiltrating eosinophils, T lymphocytes, and mast cells and releasing of pro-inflammatory cytokines and lipid mediators (Wang et al. 2021). More than 300 million individuals worldwide have asthma (Dharmage et al. 2019). According to the Global Initiative for Asthma (GINA), there might be 400 million cases of asthma by 2025 (Wang et al. 2022). The development of asthma has been associated with an imbalance between T helper (Th)1/Th2 immune responses (Zhu et al. 2020). Inappropriate, excessive Th2 response results in the increased release of Th2-related cytokines, such as interleukin(IL)-4, IL-5, and IL-13 (Zheng et al. 2019), which cause immunoglobulin E (IgE) synthesis, mucus hypersecretion, and accumulation of oxygen free radicals by the airway infiltrating eosinophils and other immune cells (Antunes et al. 2022). Allergic asthma is frequently associated with increased reactive oxygen species (ROS) formation and impaired antioxidant mechanisms, causing oxidative stress in the lungs (Zhu et al. 2021), which affects antioxidant activity, enhances the production of inflammatory mediators, and causes goblet cell hyperplasia (Malaquias et al. 2018).

The pathogenesis of asthma has been linked to several biochemical cascades, especially nuclear factor kappa beta (NF-κB) (Bai et al. 2022). NF-κB is a key mediator of immunological and inflammatory actions via stimulating target gene transcription and initiating inflammatory cytokines (Edwards et al. 2009; Zhou et al. 2014). The sustained activation of NF-κB is related to asthmatic inflammation. It has been shown that suppressing the NF-κB signaling cascade could ameliorate ovalbumin (OVA)-induced allergic asthma (Wang et al. 2018).

For the preclinical testing of asthma medications, researchers can choose from a variety of animal models of experimental asthma. OVA-induced experimental asthma is a common model for testing potential antiasthmatic drugs (Thakur et al. 2019). High levels of serum IgE, airway inflammation, epithelial hypertrophy, goblet cell hyperplasia, and airway hyperresponsiveness are reported in OVA-sensitized and challenged animals, making them a popular asthma model (Kim et al. 2019).

Corticosteroids and antileukotrienes are two principal anti-inflammatory medications for the treatment of asthma, which are successful for most patients (Ducharme 2004). Nevertheless, some people show serious asthma symptoms and resist such drugs (Woolcock 1993). Nowadays, discovering medications to treat and protect against severe asthmatic conditions is critical (Eger and Bel 2019).

Marine animals are one of the primary sources of new natural compounds with promising biological applications (Karthikeyan et al. 2022). Some marine natural products are of enormous importance because of their therapeutic significance (Papon et al. 2022). Echinochrome (Ech) is the most frequent naturally-occurring pigment found in sea urchin shells, spines, and ova, which has the most potent antioxidant properties (Mohamed 2021). Elimination of ROS, binding metal ions, and reducing lipid peroxidation are all among the antioxidant processes that Ech can use (Jeong et al. 2014). It has antioxidative, antiviral, antialgal, and antimicrobial properties (Park et al. 2019). A previous study has shown that Ech possesses antifibrotic and anti-inflammatory properties by suppressing fibroblast stimulation and proinflammatory cytokine expression (Park et al. 2021). For the first time, the current research is aimed at evaluating the effect of Ech on lung oxidative stress markers in addition to airway inflammation and remodeling.

## Materials and methods

### Chemicals and kits

Standard Ech (Vladivostok, Russia) and Dulbecco’s phosphate buffer saline (PBS) 10X were obtained from SEROX GmbH® (Mannheim, Germany). Ovalbumin, aluminum hydroxide, and other materials used in this research were obtained from Sigma-Aldrich (St. Louis, MO, USA). OVA-specific IgE (Cat No. E-20391Mo) and total IgE (Cat No. E-20550Mo) (Houston, TX, USA), IL-4 (Cat No. BMS613), IL-13 (Cat No. BMS6015), and IL-1β (Cat No. BMS6002) were obtained from Invitrogen by Thermo Fisher Scientific (USA). Rat anti-mouse PerCP-conjugated CD3+ antibody (Clone: 145-2C11) was from BD Biosciences (USA). Anti-mouse phospho-RELA (S536) polyclonal antibody (p-NFκB-p65) was from Cusabio, Biotech Co., Ltd. DAB-substrate kit was from Thermo Fisher Scientific (USA).

### Animals

Female BALB/c mice (*Mus musculus*) (6–8 weeks old) were obtained from the National Research Center (Egypt). They were housed and grouped in sterile cages and fed, and had free access to water *ad libitum*. This study was approved by the Institutional Animal Care and Use Committee (IACUC) with a number of CU/I/F/32/22 at Cairo University in Egypt. All of the experimental procedures were carried out in accordance with international standards for the care and use of laboratory animals and performed in accordance with the advice provided in the most recent edition of the Guide for the Care and Use of Laboratory Animals, National Research Council, USA.

### Ech isolation

The Amarowicz method was used to isolate the pigments in the shell and spines, with minor modifications (Amarowicz et al. 1994; Kuwahara et al. 2009). The shells and spines were cleaned in cold tepid water, dried in air for two days in the dark at 4°C, and ground after the internal organs were eliminated. The obtained powder was dissolved by the addition of 30 mL of 6 M HCl. With an equal volume of diethyl ether, the formed dark-red solution was separated four times. The obtained ether layer was treated with 5% NaCl. Then anhydrous sodium sulfate was added to remove water from the ether solution. Finally, the Heidolph rotary evaporator (Schwabach, Germany) evaporated the diethyl ether under reduced pressure. Ech was obtained and kept in the dark at −30°C.

### High-performance liquid chromatography (HPLC) analysis

A Shimadzu HPLC system (Kyoto, Japan) was used, which included two LC20AD pumps, a DGU-20 A3 degasser, and an SPD-M20 A diode-array detector. With a 1.0 mL/min flow rate, chromatographic separation was performed using a Zorbax Eclipse Plus C18 column (250 mm 4.6 mm, 5 m, Agilent Acetonitrile/methanol (5:9), and 0.1% formic acid made up the binary mobile phase. An elution profile looked like this: 30–80% acetonitrile in formic acid for 0–25 min (linear gradient). The volume of injection was 20µL. Between 200 and 800 nm, the detection was noted. The data analysis system comprised the LC Solution (Shimadzu). DMSO was used to dissolve Ech at a concentration of 5 mg/mL.

### OVA sensitization and inhalation

**Fig. 1:**
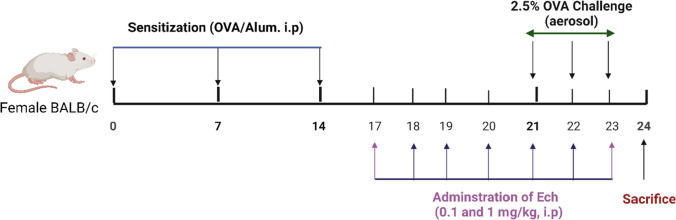
Diagram representing the protocol for the induction of the experimental model of allergic asthma along with Ech treatment

Following acclimatization for two weeks, 32 mice were randomly assigned into 4 groups (8 mice/group), including a control group, OVA group, low-dose Ech (0.1 mg/kg) group, and high-dose Ech (1 mg/kg) group. The asthma model was established according to Ou et al. (2021) with some modifications. The mice were sensitized intraperitoneally with 20 μg of OVA and 1 mg of alum gel dissolved in 200 μL of 0.9% saline on the 1st, 7th, and 14th days. From day 21 to 23, mice were placed into a container and challenged by atomization inhalation with a continuous dose of 2.5% OVA (1 hour per day). Mice from the control group were given the same volume of saline instead of OVA. One hour before challenging the mice, Ech was injected intraperitoneally for 7 days starting from day 17, while the control group was given the same amount of 2% DMSO, as illustrated in Fig. 1.

### Sample collection

At the end of the experiment, by using isoflurane, mice were anesthetized and blood was drawn from the retro-orbital plexus for measuring the allergic and inflammatory mediators in the serum. The bronchoalveolar lavage fluid (BALF) was collected for identifying and quantifying the infiltrating inflammatory cells as well as the cytokine levels. Finally, the lung was obtained for the histopathological and immunohistochemical examination.

### Evaluation of nasal scratching

Asthmatic behavior was evaluated according to Liu et al. (2022) with minor modifications. On the last day, nasal scratching was evaluated and scored for 10 min after the last challenge with 2.5% OVA. The scoring was as follows: mice who scratched their noses 0–2 times scored 0 points, 3–5 times scored 1 point, 6–8 times scored 2 points, and nine times or more scored 3 points.

### Collection of BALF and cell count

The trachea was intubated and rinsed with 0.6 mL PBS buffer containing bovine serum albumin (BSA) and EDTA three times. Each mouse’s lavage solutions were preserved on ice and then centrifuged at 2000 rpm for 10 min at 4°C using a cooling centrifuge (Sigma, 3-30K, Germany); the supernatants were gathered and stored at −80 °C. The pellet was suspended in 100 μL PBS and stained with Wright–Giemsa stain. Total and differential inflammatory cell counts were calculated. By counting 100 cells per slide at a magnification of ×40, the slides were examined for differential cell count (Wang et al. 2019).

### Measurement of OVA-specific IgE in serum and BALF

Blood was obtained from the retro-orbital plexus and then centrifuged at 3000 rpm for 15 min at 4°C to obtain serum, which was kept at −80°C. Serum and BALF levels of OVA-specific IgE were measured using ELISA kits by an ELISA plate reader (DAS Instruments, model A3, Rome, Italy).

### Determination of serum total IgE, IL-4, and IL-1β and BALF IL-13 levels

Serum levels of total IgE, IL-4, and IL-1β were measured. At the same time, the BALF supernatant was used to measure the concentration of IL-13 using ELISA kits according to the manufacturer’s procedures.

### Determination of lipid peroxidation, GSH level, and catalase and GST activities in the lung tissue

The supernatant was collected by centrifuging the lung tissues at 25000 rpm for 10 min at 4°C after weighting and homogenizing them in 0.1M Tris-HCl buffer (pH 7.4). Subsequently, the levels of malondialdehyde (MDA), nitric oxide (NO), glutathione (GSH), glutathione-S-transferase (GST), and catalase (CAT) activities were measured by commercial kits.

### Lung histology

Mice were dissected and the lung was collected and fixed in 10% neutral buffer formalin for 24 hours; then, 4-µm-thick sections were cut and stained with hematoxylin and eosin (H&E) and periodic-acid Schiff (PAS). The extent of lung inflammation and hyperplasia of goblet cells was evaluated based on a previously reported scoring system (Tanaka et al. 2001). In brief, the semi-quantitative scoring system for lung inflammation was as follows: 0, no cells; 1, a few cells; 2, a ring of cells (1 cell layer deep); 3, a ring of cells (2–4 cells deep); and 4, a ring of cells (> 4 cells deep). At the same time, the system for mucus secretion was as follows: 0, < 0.5% PAS-positive cells; 1, < 25%; 2, 25–50%; 3, 50–75%; and 4, > 75%. Four different sections were used to score infiltrating immune cells and goblet cells.

### Flow cytometric analysis

Fresh spleens were collected from each group of mice and a syringe piston was used to squeeze the samples in PBS gently. Cells were suspended in 1 mL of PBS and centrifuged for 10 min at 1300 rpm at 4°C. After discarding the supernatants and gently vortexing the pellets in 200 μL of PBS, 100 μL of suspended cells was put into all reaction tubes, then 3 μL rat anti-mouse PerCP-conjugated CD3+ antibody. After that, the reaction tubes were placed in the dark for 20 min, and 2 mL of the lysis buffer was added and placed again in the dark for 10 min. For 3 min, tubes were centrifuged. The pellet was rinsed with 1 mL of PBS supplemented with BSA and EDTA for washing after the supernatants were discarded and centrifuged. Finally, cell pellets were resuspended in 400 μL of PBS, and the supernatants were discarded before the analyses using FACS Melody (BD Biosciences, USA).

### Immunohistochemistry (IHC)

Tissue sections were cut into 4-µm sections, de-paraffinized, rehydrated, and exposed to heat-induced antigen retrieval step for 15 min, followed by blocking steps for protein and endogenous peroxidases using BSA and hydrogen peroxide, respectively. After washing in PBS, tissue slides were incubated with anti-mouse phospho-RELA (S536) polyclonal antibody (p-NFκB-p65) (at a dilution of 1:200) for 12 h at the refrigerator; then HRP-labeled secondary antibody was applied. After washing, the DAB substrate was utilized to produce the color. Positive expression was assessed as area % using ImageJ software.

### Statistical analysis

Statistical Package for the Social Sciences (SPSS) was utilized for statistical analyses (IBM). Mean ± SEM was used to express values. The differences between groups were evaluated using one-way analysis of variance (ANOVA). Graphs were drawn using the software of GraphPad Prism version 8. The analysis of flow cytometry data was performed using BD FACSDiva software version 6.1.1. Duncan’s post hoc test was employed to compare the group means, and *P* < 0.05 was regarded as statistically significant.

## Results

### HPLC data

**Fig. 2:**
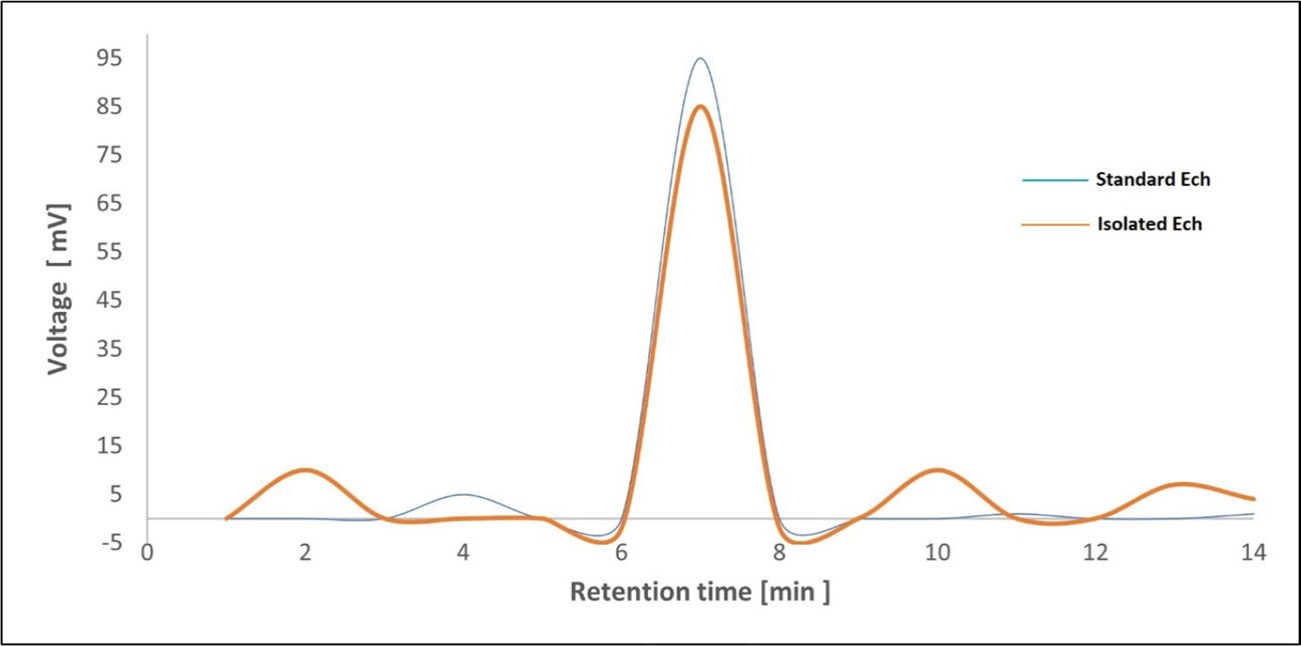
HPLC chromatograph of standard Ech and isolated Eche from sea urchin

As shown in Fig. 2, the HPLC analyses of isolated Ech revealed a significant peak with a retention period of 7.11 min that matched the standard Ech with a total concentration of 85.02%.

### Ech attenuated nasal scratching and the infiltration of inflammatory cells in BALF

Shortness of breath, wheezing, and sneezing are common clinical signs of asthma. Although spotting these symptoms in rodents can be difficult, they show signs like scratching, tickling, and rapid breathing (Wang et al. 2015). It was noticed that OVA-challenged mice had significantly elevated overall nasal scratching scores. However, following Ech treatment, the scores significantly decreased, showing the ameliorative effect of Ech on asthma symptoms. Additionally, the inflammatory cell counts in the BALF of the OVA group were examined to study the impact of Ech on the infiltrating immune cells. The OVA group’s infiltrating inflammatory cells in BALF were notably greater than in the control group. Ech treatment (0.1 and 1 mg/kg) inhibited this infiltration in a dose-dependent way. These findings are shown in Table 1.

**Table 1:** Ech effects on nasal scratching scoring and BALF infiltering inflammatory cells of OVA-challenged mice

Group	Nasal scratching score	Total cell count (×10^6^/mL)	Eosinophil count (×10^5^/mL)	Neutrophil count (×10^5^/mL)	Lymphocyte count (×10^5^/mL)	Macrophage count (×10^5^/mL)
Control	0.75 ± 0.25^a^	0.80 ± 0.12^a^	0.00 ± 0.00^a^	1.60 ± 0.23^a^	2.00 ± 0.29^a^	4.40 ± 0.64^a^
OVA	2.50 ± 0.29^b^	10.67 ± 0.67^d^	55.00 ± 2.52^d^	21.13 ± 2.77^d^	17.00 ± 2.08^c^	13.53 ± 1.78^b^
Ech (0.1mg/kg)	1.50 ± 0.29^a^	7.25 ± 0.08^c^	37.37 ± 4.00^c^	13.30 ± 1.26^c^	6.06 ± 1.24^ab^	15.78 ± 3.38^b^
Ech (1 mg/kg)	1.25 ± 0.25^a^	4.60 ± 0.31^b^	13.99 ± 0.59^b^	7.39 ± 1.77^b^	8.50 ± 0.76^b^	16.10 ± 1.07^b^

Values are given as means for 8 mice in each group ± standard error of the mean (SEM). The value that does not share a common superscript letter is significantly different (*P* <0.05). The values are arranged from the lowest (a) to the highest (d). The difference between groups is (*P* <0.05)

### Ech decreased the serum and BALF levels of immunoglobulins and cytokines

The key characteristic of allergic asthma is a rise in blood IgE levels (Scirica et al. 2007). In asthmatic mice, the levels of IgE, OVA-specific IgE, IL-4, IL-1β, and IL-13 increased significantly (*P* <0.05). While Ech treatment dose-dependently decreased these mediators’ levels compared to the asthmatic group, as shown in Table 2.

**Table 2:** The effect of Ech on serum and BALF concentrations of IgE, OVA-specific IgE, IL-4, IL-1β, and IL-13 of OVA-challenged mice

Groups	Serum OVA-specific IgE (OD)	BALF OVA-specific IgE (OD)	Serum IgE (pg/mL)	Serum IL-4 (pg/mL)	Serum IL-β (pg/mL)	BALF IL-13 (pg/mL)
Control	0.08±0.01^a^	0.10±0.01^a^	318.60 8.71^a^	13.41±8.51^a^	91.78±1.98^a^	19.17±2.54^a^
OVA	0.44±0.01^d^	0.44±0.01^d^	670.60±53.44^c^	210.18±47.97^c^	120.67±2.53^d^	73.78±16.05^b^
Ech (0.1 mg/kg)	0.39±0.01^c^	0.35±0.01^c^	498.60±10.20^b^	93.41±7.94 ^b^	110.67±0.83^c^	48.77±4.20^a^
Ech (1 mg/kg)	0.29±0.02^b^	0.32±0.01^b^	430.60±17.20^b^	52.24±6.34 ^ab^	102.15±2.08^b^	29.01±2.46^a^

Values are given as means for 8 mice in each group ± standard error of the mean (SEM). The value that does not share a common letter superscript is significantly different (*P* <0.05*).* The values are arranged from the lowest (a) to the highest (d). The difference between groups is (*P* <0.05)

### Effect of Ech on oxidative stress parameters

Table 3 provides data on the lung oxidative stress markers (MDA, NO, GSH, CAT, and GST). It was found that OVA mice had significantly greater MDA and NO concentrations than the control group (*P* <0.05). In contrast, a significant decline (*P* <0.05) was shown in the Ech-treated group in a dose-dependent manner. Additionally, there was a significant decline (*P* <0.05) in the concentration of GSH and GST and CAT activities in asthmatic mice. Despite this, a significant rise (*P* <0.05) was noticed in the Ech-treated mice in a dose-dependent way.

**Table 3:** Ech effects on lung homogenate oxidative stress parameters of OVA-challenged mice

Groups	MDA (nmol/g·tissue)	NO (µmol/g·tissue)	GSH (mg/g·tissue)	CAT (U/g·tissue)	GST (U/g·tissue)
Control	5.86±0.23^a^	508.54±20.59^a^	0.48±0.03^d^	51.50±4.55^d^	3.81±0.19^c^
OVA	8.30±0.22^c^	738.42±27.22^c^	0.08±0.02^a^	12.17±1.07^a^	2.19±0.12^a^
Ech (0.1 mg/kg)	7.32±0.51^b^	632.46±37.73^b^	0.17±0.02^b^	22.57±0.91^b^	3.06±0.21^b^
Ech (1 mg/kg)	6.21±0.15^a^	537.68±30.73^a^	0.37±0.04^c^	37.57±2.80^c^	3.67±0.17^c^

Values are given as means for 8 mice in each group ± standard error of the mean (SEM). The value that does not share a common superscript letter is significantly different (*P* <0.05*).* The values are arranged from the lowest (a) to the highest (d). The difference between groups is (*P* <0.05)

### Ech ameliorated pathological alterations in lung tissue

**Fig. 3:**
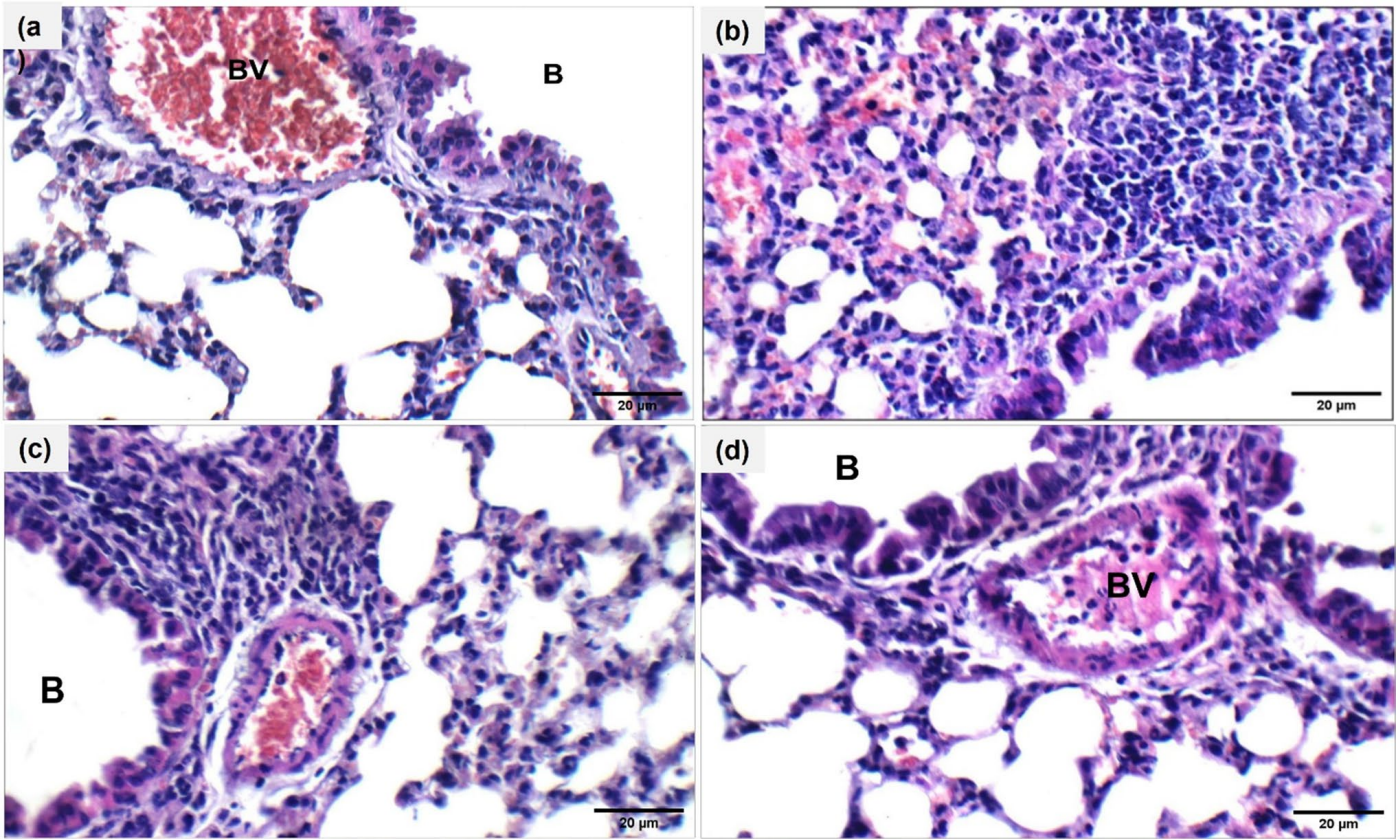
Effect of Ech on histopathological alterations in the lung tissue of mice of different groups, (H&E) (400X). **a** Control: the lung showed average bronchioles (B) with average epithelial lining and average blood vessels (BV). **b** OVA: the lung showed bronchioles with ulcerated epithelial lining, mildly congested blood vessels, thickened alveolar walls, and excessive peri-bronchiolar inflammatory infiltrate with scattered eosinophils. **c** 0.1 mg/kg Ech: bronchioles with average epithelial lining and increased peri-bronchiolar and peri-vascular inflammatory infiltrate and average blood vessels. **d** 1 mg/kg Ech: bronchioles with average epithelial lining, average blood vessels, average alveolar walls, and mild peri-bronchiolar and peri-vascular inflammatory infiltrate

**Fig. 4:**
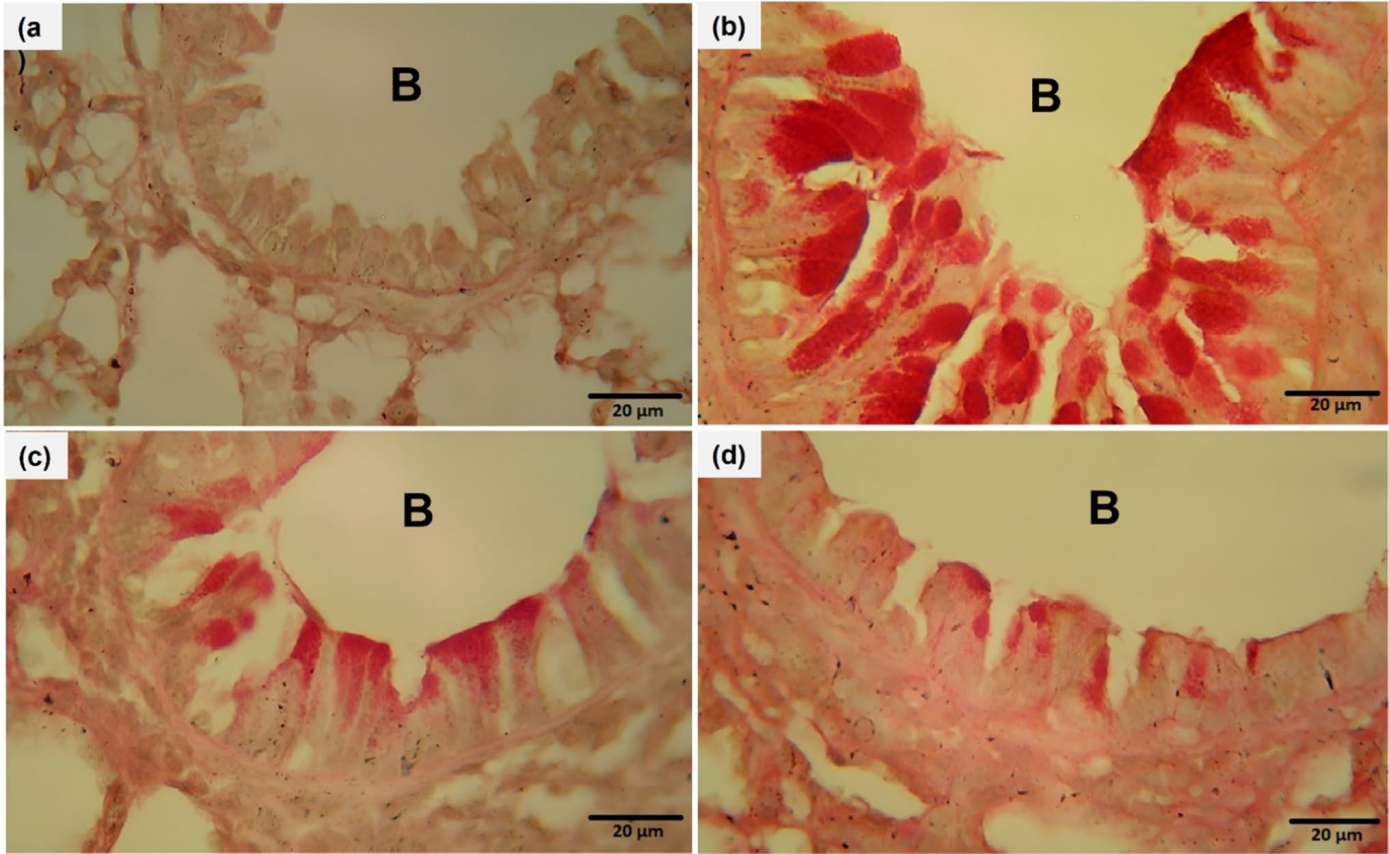
Effect of Ech on mucus secretion in the lung tissue of mice of different groups, (H&E) (400X). **a** Control, **b** OVA, **c** 0.1 mg/kg Ech, and **d** 1 mg/kg Ech groups. B= Bronchioles

H&E and PAS were used to demonstrate airway infiltrating immune cells, goblet cell hyperplasia, and mucus secretion. The OVA group’s lung tissue displayed greater immune cell infiltration into the airways (Fig. 3), excessive mucus production, and goblet cell hyperplasia (Fig. 4). However, Ech treatment improved these changes in a dose-dependent way. The inflammation and mucus scores are shown in Table 4.

**Table 4:** Ech effects on inflammation and mucus score of the lung tissue of different groups

Group	Inflammation score	Mucus score
Control	0.67 ± 0.33^a^	0.0 ± 0.0^a^
OVA	3.67 ± 0.33^c^	3.33 ± 0.33^c^
Ech (0.1 mg/kg)	2.33 ± 0.33^b^	1.67 ± 0.33^b^
Ech (1 mg/kg)	1.33 ± 0.33^ab^	0.67 ± 0.33^a^

Values are given as means for 8 mice in each group ± standard error of the mean (SEM). The value that does not share a common superscript letter is significantly different (*P* <0.05*).* The values are arranged from the lowest (a) to the highest (d). The difference between groups is (*P* <0.05)

### Effect of Ech on the percentage of CD3^+^ cells

**Fig. 5:**
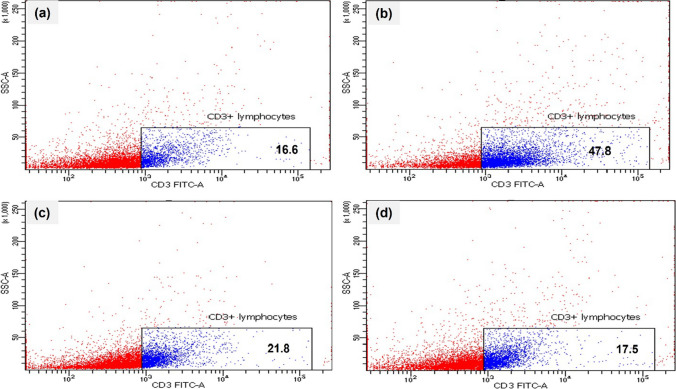
Dot plots representing the percentage of CD3^+^ cells in the spleen of mice of different groups. **a** Control, **b** OVA, **c** 0.1 mg/kg Ech, and **d** 1 mg/kg Ech groups

The percentage of CD3+ cells in asthmatic mice increased significantly (*P* <0.05) compared to the control group. However, a significant decline (*P* <0.05) was shown after Ech administration in a dose-dependent way (Fig. 5) (Table 5).

**Table 5:** Ech effect on the percentage of CD3+ lymphocyte was determined in the lung tissue of different animal groups

Group	% Frequency of CD3^+^ lymphocytes
Control	16.77 ± 0.55^a^
OVA	47.53 ± 10.68^b^
Ech (0.1mg/kg)	26.13 ± 4.91^a^
Ech (1 mg/kg)	22.00 ± 1.04^a^

Values are given as means for 8 mice in each group ± standard error of the mean (SEM). The value that does not share a common superscript letter is significantly different (*P* <0.05*).* The values are arranged from the lowest (a) to the highest (d). The difference between groups is (*P* <0.05)

### Immunohistochemical detection of phosphorylated-NF-κB p65

**Fig. 6:**
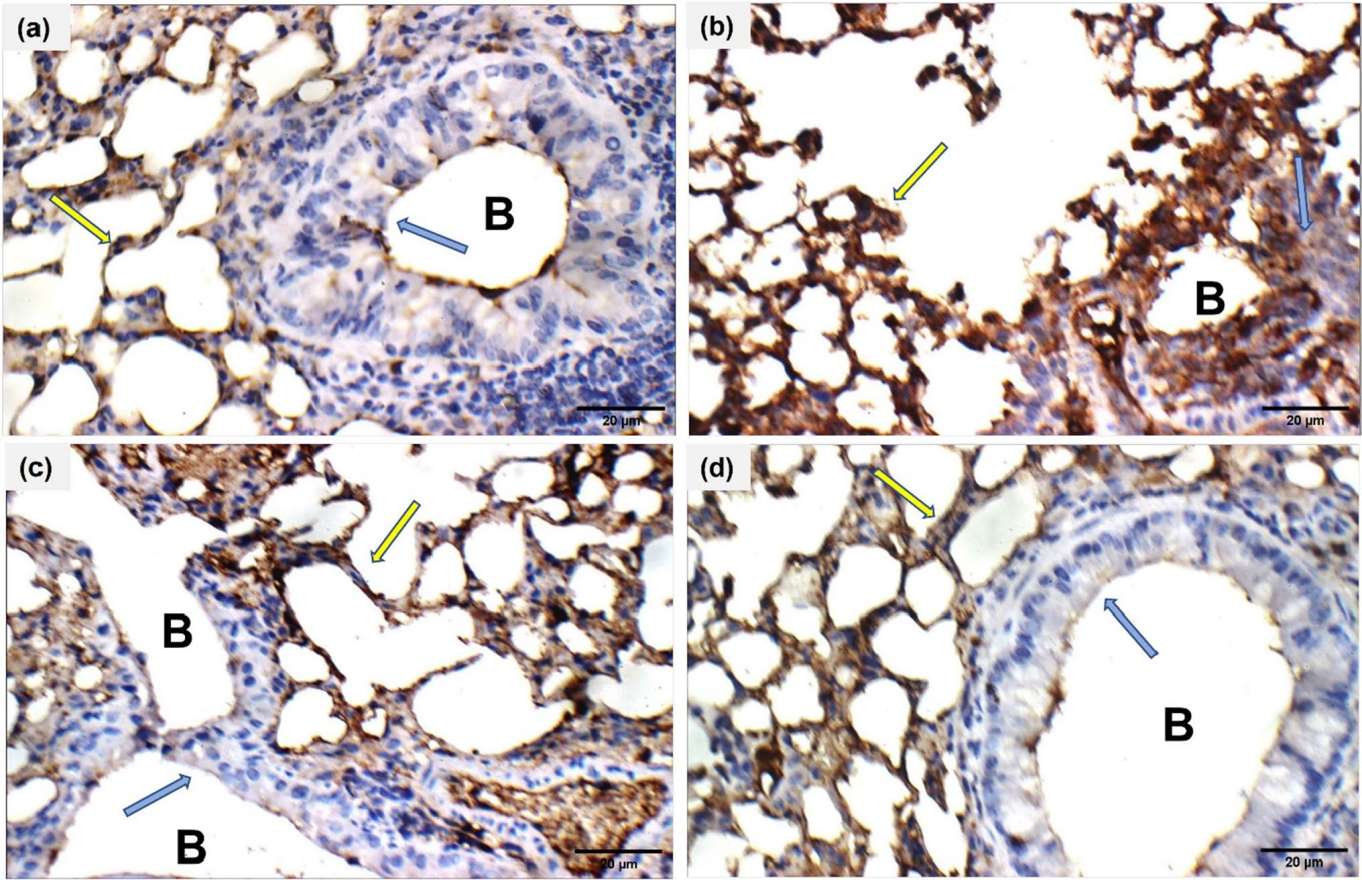
Immunohistochemistry of Phospho-NF-κB p65 expression in lung tissue (400X) both in the bronchioles (B) (blue arrow) and the alveoli (yellow arrow). **a** Control, **b** OVA, **c** 0.1 mg/kg Ech, and **d** 1 mg/kg Ech groups

The immune expression of Phospho-NF-κB p65 is illustrated in Fig. 6. The lung p-NF-κB p65 area % of asthmatic mice increased significantly (*P* <0.05). However, a significant reduction (*P* < 0.05) was noticed after Ech treatment in a dose-dependent way (Table 6).

**Table 6:** Ech effect on the immunohistochemical expression of Phospho-NF-κB p65 in the lung tissue of different groups

Group	p-NF-κB p65 area %
Control	1.40 ± 0.07^a^
OVA	15.21 ± 0.48^c^
Ech (0.1 mg/kg)	3.15 ± 0.40^b^
Ech (1 mg/kg)	2.20 ± 0.12^ab^

Values are given as means for 8 mice in each group ± standard error of the mean (SEM). The value that does not share a common superscript letter is significantly different (*P* <0.05*).* The values are arranged from the lowest (a) to the highest (d). The difference between groups is (*P* <0.05)

## Discussion

Asthma is a persistent disorder of the bronchi. It is characterized by the accumulation of the airways by inflammatory cells and their secreted mediators, such as cytokine and chemokines, which cause inflammation, hyperreactivity, and irreversible airway blockage (Jung et al. 2008). Nowadays, corticosteroids are frequently used to manage asthma symptoms. However, their use is restricted due to resistance and severe problems. Additionally, this approach negatively impacts children’s bone mass and growth (Jassal 2015). Consequently, it is vital to investigate new potential therapeutic strategies as well as the underlying molecular pathophysiology of asthma. Animal models of OVA-induced airway inflammation share various cellular and molecular characteristics with human asthma (Aun et al. 2017). We examined the anti-inflammatory and antioxidant effects of Ech in an asthmatic OVA mouse model. Additionally, we evaluated behavioral, biochemical, histopathological, and immunohistopathological analyses. The effects of Ech on oxidative stress markers, antioxidant parameters, Th1 and Th2-related cytokines, IgE, infiltrating inflammatory cells, airway remodeling, and NF-κB activation were examined.

The pathophysiology of asthma was strongly influenced by Th2 cytokines such as IL-4, IL-5, and IL-13. These cytokines strongly correlate with inflammatory cell infiltration, IgE generation, eosinophil activation, and airway hyperresponsiveness (Ray and Cohn 1999). One of the most crucial cytokines for controlling Th2 inflammatory responses is IL-4, which stimulates the maturation of B cells and the switch to IgE (Renz et al. 1995). IL-13 is an essential immunomodulatory cytokine in the pathophysiology of bronchial inflammation. In terms of bronchial hyperresponsiveness and secretion of mucus, IL-13 is more significant than IL-4. Thus, inhibiting these Th2 cytokines may reduce allergic asthma (Barnes 2001). It was reported that Ech suppressed IL-4 and IL-13 in the atopic dermatitis model. In our investigation, mice treated with OVA had significantly greater levels of such cytokines, whereas Ech administration greatly reduced their production. Following OVA sensitization and inhalation, the serum and the BALF concentrations of OVA-specific IgE increased dramatically in the OVA group. At the same time, this rise was reduced in the Ech treatment groups. These outcomes are consistent with earlier research where OVA exposure raised IgE concentrations (Eftekhar et al. 2019). IL-1β induces the polarization of Th2, which activates infiltrating eosinophils and produces cytokines such as IL-5 (Rajizadeh et al. 2019). The concentration of IL-1β increased in the OVA-challenged group, while a significant decrease was shown after Ech administration. These results are consistent with previous results where the expression of IL-1β genes was significantly elevated in the OVA murine model of asthma (shakerinasab et al. 2022).

The development of asthma is highly affected by the infiltration of inflammatory cells (Chung 1986). Eosinophils and lymphocytes are among the inflammatory cells infiltrating the airways during OVA-induced asthma (Song et al. 2008). Our findings in this investigation showed that the infiltrating immune cells count was elevated in the OVA-challenged mice. In contrast, Ech treatment significantly reduced their infiltration. Although macrophage infiltration did not change, this could mean that Ech treatment influences the polarization of macrophage rather than the infiltration. These results can be supported by the study conducted by Oh et al. (2019) in which Ech administration stimulated the polarization of macrophages toward the M2 type, which helps to reduce inflammation and promote tissue repair. Additionally, flow cytometric analysis showed increased proliferation of CD3+ cells in the spleen of asthmatic mice. However, Ech treatment causes a notable decline in a dose-dependent manner.

Oxidative stress is a key mediator in the pathophysiology of asthma (Adam-Bonci et al. 2021). Previous studies showed that OVA-induced animals’ airways generate high ROS levels (Nishida et al. 2002). The accumulation of these ROS causes airway hyperresponsiveness (Sadeghi-Hashjin et al. 1996). MDA generation is one of the most critical determinants of oxidative stress. In this research, it was figured out that there was a significant rise in the MDA concentration in the OVA group. At the same time, a significant decrease was shown in the Ech-treated groups. These findings agree with the results investigated by Sadek et al. (2022), where the MDA level increased in septic rats, and Ech administration showed a significant decline in the MDA level.

NO is a potent free radical that combines with superoxide anions to generate peroxide nitrite, which can harm cell membranes (Malaquias et al. 2018). It may have a dual function: at normal concentrations, it is critical for signal transduction, and elevated NO and reactive nitrogen species levels can cause cell destruction (Yu et al. 2018). In our work, the lung tissue homogenate NO level was significantly elevated in the OVA-challenged mice. However, the Ech administration resulted in a statistically significant decline in NO level. Our findings are consistent with those made by Dweik et al. (2001), where the OVA challenge raised the level of NO in asthmatic airways.

GSH and CAT are essential antioxidants for alleviating lung cell fibrosis and damage in asthmatic patients (Rogers and Cismowski 2018). According to Martínez-Martos et al. (2014), GSH is a crucial biological antioxidant that inhibits the generation of free radicals. Generally, a reduction in GSH concentration reflects an elevation in ROS. In the study, GSH concentrations were decreased notably in the OVA group. At the same time, a significant rise was shown in the Ech treatment groups. Previous research indicated that OVA administration decreased GSH levels, which were then correlated to their deletion as a result of lipid peroxidation and ROS generation (Xiao et al. 2023). CAT can alleviate cell damage by converting H_2_O_2_ into water and oxygen (Sahiner et al. 2018). In this study, the activity of CAT was significantly reduced in OVA-challenged mice, while its activity was enhanced after Ech treatment. These results agree with the previous study (Dalouchi et al. 2021), where the CAT activity was reduced in the OVA-induced animal model. GSTs are essential in detoxifying various chemicals by combining the electrophilic molecules with GSH (Economopoulos and Sergentanis 2010). In this study, the activity of GST decreased significantly in asthmatic mice. However, a significant elevation in its activity was observed after Ech administration. These findings agree with a previous study where asthmatic mice’s lungs had reduced GST activity levels (Ajayi et al. 2022). In this research, we investigated the ameliorative effect of Ech against parameters associated with oxidative stress; this was achieved by observing changes in the production of MDA and NO as well as the levels of GSH, CAT, and GST. Our findings imply that Ech can efficiently attenuate oxidative stress and the harm it causes to the airways.

The lung histopathology results, which showed that OVA-challenged mice had higher scores for inflammation and mucus secretion than the control group, supported the biochemical findings. After Ech administration, these scores significantly decreased, and the histopathological changes were also attenuated. In another study, it was discovered that administering Ech reduced the level of oxidative stress caused by lipopolysaccharide in the lungs and inhibited airway lymphocyte infiltration (Kuznetsova et al. 2019). NF-κB plays a crucial role in the regulation of cytokine production (Huang et al. 2015). Previous research revealed that NF-κB activation contributed to airway inflammation in human and murine asthmatics (Bureau et al. 2000). The bronchial epithelium of asthmatic mice displayed a significant and rapid NF-κB p65 nuclear translocation (Zhang et al. 2015). It has been demonstrated that OVA-challenged mice had higher levels of phospho-NF-κB p65 (Zhang et al. 2017). The present immunohistochemical findings reveal that Ech significantly reduced the level of phospho-NF-кBp65. These results suggest that Ech helps treat asthma.

## Conclusion

**Fig. 7:**
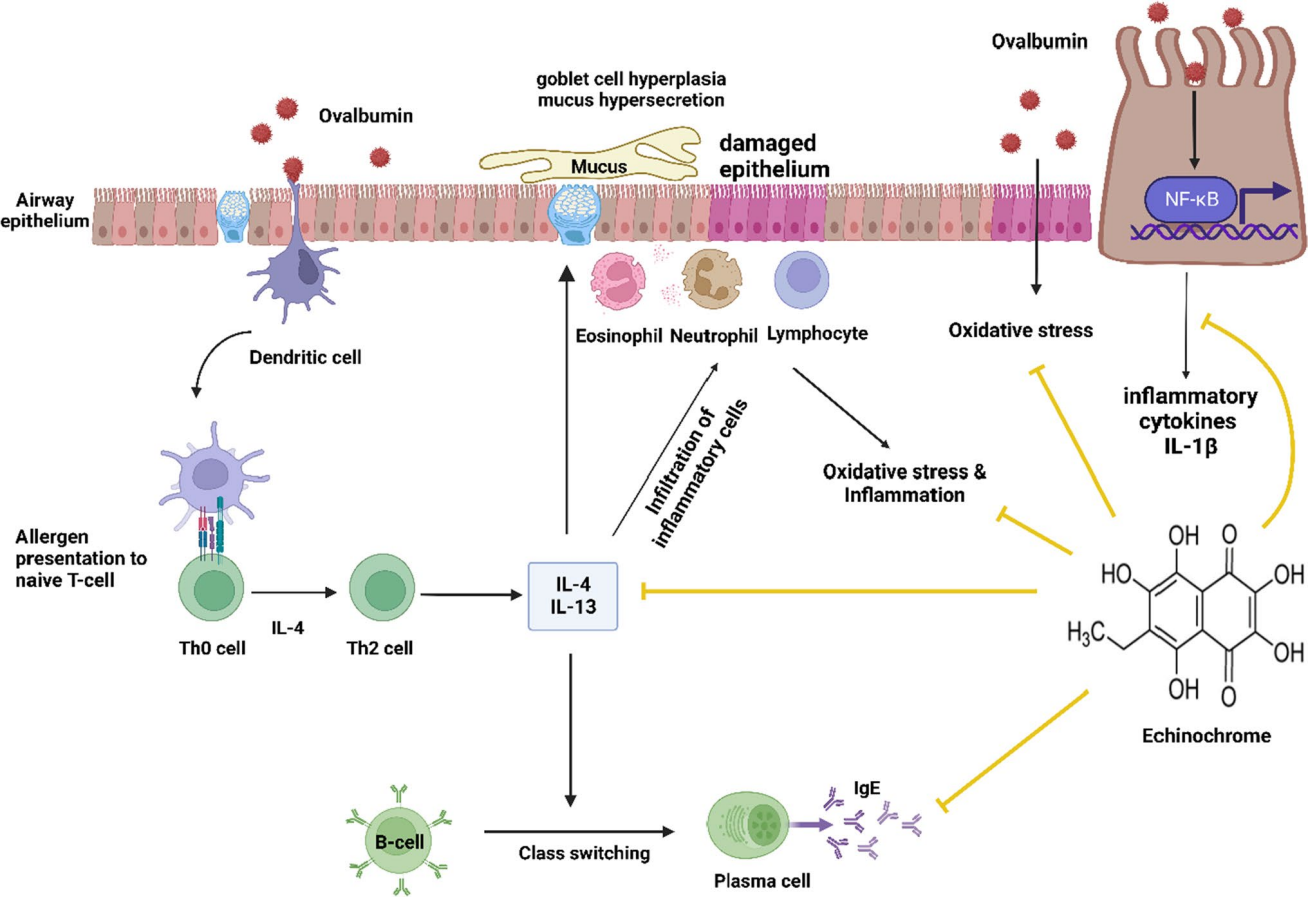
Schematic diagram showing the suggested mechanism of Ech action on airway inflammation and oxidative stress in the OVA-induced asthma model

In conclusion, our findings as shown in Fig. 7 are the first to reveal that Ech effectively protects the lungs from oxidative stress and inflammation and that this is highly dependent on restoring the balance between ROS and antioxidants, improving airway remodeling, reducing mucus secretion and goblet cells hyperplasia, and suppressing Th2-related cytokines as well as IgE and OVA-specific IgE production. Moreover, Ech administration inhibited the phosphorylation of NF-κB P65. These findings imply that it may be an effective and helpful treatment for allergic asthma.

## Data Availability

The authors confirm that the data supporting the findings of this study are available within the article. Raw data will be made available on request.
